# *MFAP2* Promotes Glioblastoma Malignant Phenotypes via Autophagy-Dependent Activation of Wnt/β-Catenin Signaling

**DOI:** 10.3390/biomedicines14051003

**Published:** 2026-04-28

**Authors:** Peihao Yang, Demeng Liu, Jiyao Wang, Chao Liu, Yan Fang

**Affiliations:** 1 Department of Pharmacy, The Second Affiliated Hospital of Zhengzhou University, Academy of Medical Sciences, Zhengzhou University, Zhengzhou 450053, China; yangpeigao0517@gs.zzu.edu.cn (P.Y.); liudemengsj@163.com (D.L.); 2 Institute of Clinical Pharmacology, School of Basic Medical Sciences, Zhengzhou University, Zhengzhou 450053, China; wwang_jiyao@126.com

**Keywords:** microfibrillar-associated protein 2, glioblastoma, autophagy, Wnt/β-catenin pathway, prognosis

## Abstract

**Background**: Microfibrillar-associated protein 2 (*MFAP2*) is implicated in various malignancies, yet its specific role and molecular mechanisms in glioblastoma (GBM) progression remain poorly understood. **Methods**: We analyzed *MFAP2* expression in human clinical specimens and murine models. Functional impacts were assessed in U251 cells via gain- and loss-of-function assays. Mechanistic studies explored the interplay between autophagic flux and Wnt/β-catenin signaling. An orthotopic GL261 syngeneic orthotopic model validated these findings in vivo. **Results**: *MFAP2* was significantly overexpressed in GBM, correlating with poor patient prognosis. In vitro, *MFAP2* markedly enhanced U251 viability, migration, and invasion while suppressing apoptosis. Mechanistically, *MFAP2* triggered autophagic flux, subsequently activating the Wnt/β-catenin cascade and its downstream targets (MMP9, c-Myc, Cyclin D1). Pharmacological inhibition of either autophagy or Wnt signaling effectively abrogated these oncogenic phenotypes. In vivo, *MFAP2* knockdown reduced tumor volume by 62.4% and suppressed the autophagy–Wnt axis. **Conclusions**: *MFAP2* is an oncogenic regulator in glioblastoma models that links autophagy activity to Wnt/β-catenin signaling. Our findings support *MFAP2* as a candidate prognostic biomarker and a potential therapeutic target; however, additional validation in larger molecularly annotated clinical cohorts and multiple GBM models is warranted.

## 1. Introduction

Glioblastoma (GBM) stands as the most aggressive and lethal primary brain tumor in adults, defined by relentless proliferation, pervasive invasiveness, near-universal recurrence, and profound resistance to conventional radiotherapy and chemotherapy [1]. Despite incremental advances in surgical resection, radiotherapy, and targeted pharmacotherapy, the prognosis for GBM patients remains uniformly dismal [2]. Median overall survival hovers at just 15–16 months, and late-stage patients endure severely compromised quality of life due to treatment-refractory tumor recurrence [3]. These clinical imperatives underscore an urgent need to decode the molecular circuitry driving GBM progression and identify actionable therapeutic vulnerabilities.

Microfibrillar-Associated Protein 2 (*MFAP2*), an extracellular matrix (ECM)-embedded glycoprotein historically linked to microfibril assembly and tissue homeostasis [4], has emerged as a promising oncogenic mediator across multiple cancer types. Pan-cancer transcriptomic analyses reveal consistent *MFAP2* overexpression in malignant tissues, with elevated levels correlating with advanced tumor stage, higher histological grade, and inferior patient survival [5]. In glioblastomas specifically, *MFAP2* expression is upregulated 3.3-fold relative to normal brain tissue, and high *MFAP2* levels associate with aggressive clinicopathological features and poor prognosis [6]. These observations position *MFAP2* as a putative driver of glioblastoma tumorigenesis, though its functional role and mechanistic underpinnings in GBM remain entirely uncharacterized.

Mechanistic investigations in other malignancies have begun to unravel *MFAP2*’s oncogenic properties. In melanoma, *MFAP2* promotes cell migration and invasion by activating the Wnt/β-catenin pathway, thereby inducing epithelial–mesenchymal transition (EMT) [7]. In oral squamous cell carcinoma (OSCC), *MFAP2* drives Wnt/β-catenin signaling through an autophagy-dependent mechanism; silencing *MFAP2* reduces β-catenin and its downstream effectors (MMP9, c-Myc, Cyclin D1, Survivin), while autophagy induction restores this signaling axis [8]. These findings suggest *MFAP2* may orchestrate tumor progression through coordinated regulation of ECM dynamics, autophagy, and Wnt signaling.

The Wnt/β-catenin pathway is a well-established driver of GBM pathogenesis, with aberrant activation promoting proliferation, invasion, therapeutic resistance, and stem cell maintenance [9]. Wnt ligands such as Wnt1 and Wnt3a are frequently overexpressed in GBM, and genetic or pharmacological inhibition of Wnt signaling reduces tumor growth and extends survival in preclinical models [10]. Concurrently, autophagy—a lysosome-dependent degradation pathway critical for cellular homeostasis—exerts context-dependent effects on GBM progression, regulating cell survival, therapy resistance, and phenotypic plasticity. Emerging evidence indicates bidirectional crosstalk between Wnt signaling and autophagy in GBM: Wnt/β-catenin activation induces autophagy via upregulation of ATG9B, while Wnt inhibition suppresses autophagy and enhances chemotherapy sensitivity [11]. This complex interplay highlights the need to identify upstream regulators that coordinate these pathways in GBM.

Despite these advances, the extracellular signals linking ECM components like *MFAP2* to the Wnt/autophagy axis in GBM remain a critical knowledge gap. Emerging evidence underscores that the ECM is not merely a static structural scaffold, but a dynamic microenvironment that actively orchestrates intracellular survival programs—including autophagic flux—to drive tumor progression and therapeutic resistance [12]. However, the specific ECM sensors and mediators that couple the extracellular microenvironment to intracellular autophagy in GBM remain insufficiently defined.

The major innovative aspect of our study lies in shifting the paradigm of *MFAP2* from a passive structural component to an active, upstream oncogenic driver in GBM. Building on our preliminary findings that *MFAP2* is overexpressed in GBM and correlates with poor prognosis, we hypothesized that *MFAP2* links the extracellular microenvironment to intracellular signaling cascades by activating the Wnt/β-catenin pathway through an autophagy-dependent mechanism.

To rigorously test this hypothesis and bridge the aforementioned knowledge gap, the specific objectives of this study were fourfold: to validate the clinical relevance and prognostic value of *MFAP2* upregulation using public transcriptomic datasets and patient specimens; to characterize the functional role of *MFAP2* in modulating GBM cell proliferation, survival, and migratory-invasive behaviors in vitro; to mechanistically decode the novel *MFAP2*–autophagy–Wnt/β-catenin signaling axis using targeted pharmacological and genetic perturbations; and to confirm the pro-tumorigenic role of *MFAP2* in vivo using an immunocompetent syngeneic orthotopic GL261 model. By explicitly elucidating this novel ECM-to-signaling coupling, this study provides original mechanistic insights into GBM biology and highlights MFAP2 as a promising biological vulnerability and candidate therapeutic target.

## 2. Materials and Methods

### 2.1. Bioinformatics and Data Acquisition

Normal and cancerous tissue datasets were retrieved from the GTEx database (https://gtexportal.org), accessed on 17 April 2025, and The Cancer Genome Atlas (TCGA; https://portal.gdc.cancer.gov), accessed on 18 April 2025, respectively. Data were analyzed using R software (version 4.0.3; The R Foundation for Statistical Computing, Vienna, Austria), utilizing packages including ‘stats’ and ‘car’ for statistical analysis and ‘ggplot2’ for data visualization. This analysis focused on the differential expression of MFAP2 across different cancer types and normal tissues.

### 2.2. Patient Samples

Tissue samples, including six GBM specimens and six adjacent non-tumorous tissues, were obtained from patients undergoing surgery at the Second Affiliated Hospital of Zhengzhou University (Zhengzhou, China). The collection and use of clinical samples were approved by the Ethics Committee of the Second Affiliated Hospital of Zhengzhou University (Approval No. KY2025120) and conducted in accordance with the principles of the Declaration of Helsinki. Written informed consent was obtained from all participants prior to sample collection.

### 2.3. Cell Culture

The U251 and U87MG human glioblastoma cell line was purchased from the American Type Culture Collection (ATCC; Manassas, VA, USA). Cells were cultured in high-glucose Dulbecco’s modified Eagle’s medium (DMEM) supplemented with 10% fetal bovine serum (FBS) and 1% penicillin/streptomycin (Gibco; Thermo Fisher Scientific, Inc., Waltham, MA, USA), and maintained at 37 °C in a humidified incubator with 5% CO_2_.

### 2.4. Cell Transfection

To knock down *MFAP2*, a small interfering RNA (siRNA) targeting *MFAP2* (si-*MFAP2*: 5′-CCAUACACAGGCCUUGCAUGCAATT-3′ and 5′-UUGCAAGGCCUGUGUGUGGAT-3′) and the corresponding negative control (si-NC: 5′-UUCUCCGAACGUCGUCUGGAGUT-3′) were designed and synthesized by Sangon Biotech Co., Ltd. (Shanghai, China). For *MFAP2* overexpression, the *MFAP2* overexpression plasmid (pcDNA3.1(+)-*MFAP2*; oe-*MFAP2*) and the empty control vector were also purchased from Sangon Biotech Co., Ltd. One day prior to transfection, U251 and U87MG cells (2 × 10^5^ cells/well) were seeded into 6-well plates and cultured until they reached 50–70% confluence. Transfection was performed using si-RNA-Mate (Sangon Biotech Co., Ltd.) according to the manufacturer’s instructions. Cells were maintained in a humidified incubator at 37 °C with 5% CO_2_. The transfection mixture was replaced with fresh DMEM 6 h post-transfection. Cells were harvested for subsequent experiments 24 h after transfection.

### 2.5. Cell Counting Kit-8 (CCK-8) Assay

U251 and U87MG cells (cells 2 × 10^3^ cells/mL) were seeded into 96-well plates and cultured for 24 h prior to transfection. At 24 h post-transfection, 10 µL of CCK-8 solution (GLPBIO, Montclair, CA, USA) was added to each well, and the plates were incubated at 37 °C for 1 h. Cell viability was determined by measuring the absorbance at 450 nm using a microplate reader (Bio-Rad Laboratories, Inc., Hercules, CA, USA) [8].

### 2.6. Flow Cytometry Analysis

At 24 h post-transfection, U251 and U87MG cells were detached using trypsin without EDTA (KeyGen Biotech, Nanjing, China). Subsequently, 5 × 10^5^ cells were resuspended in 1 mL of 1X Annexin binding buffer. A 100-µL aliquot of the cell suspension was transferred to a 1.5 mL conical tube containing 5 µL of FITC-conjugated Annexin V and 5 µL of propidium iodide (PI). The suspension was gently mixed and incubated in the dark at room temperature for 10 min. Apoptosis was assessed using the BD Pharmingen™ FITC Annexin-V Apoptosis Detection Kit (KeyGen Biotech). The binding of FITC-Annexin V and PI was analyzed using a FACSCalibur flow cytometer (BD Biosciences, Franklin Lakes, NJ, USA) with CellQuest software (version 5.1; BD Biosciences) [13].

### 2.7. Wound Healing and Transwell Assays

For the wound healing assay, U251 and U87MG cells (2 × 10^5^ cells/mL) were seeded into 6-well plates and cultured for 24 h until reaching confluence. A wound was created by scraping the confluent cell monolayer with a 200-µL pipette tip. Images of the wound were captured under a light microscope (Olympus Corp., Tokyo, Japan) immediately after scratching (0 h). Wound closure was assessed after 24 and 48 h of incubation in serum-free medium (2 mL per well) at 37 °C [8]. For the Transwell invasion assay, 24 h post-transfection, U251 and U87MG cells (1 × 10^5^ cells/mL) were resuspended in 200 µL of serum-free DMEM and added to the upper chamber of Transwell inserts (8-µm pore size; Corning, Inc., Corning, NY, USA) pre-coated with Matrigel. Subsequently, 600 µL of complete medium containing 10% FBS was added to the lower chamber. After 48 h of incubation, non-migrated cells were carefully removed from the upper surface of the membrane. The invaded cells on the lower surface were fixed with 4% paraformaldehyde for 10 min and stained with 0.1% crystal violet for 30 min. Images of invaded cells were captured under a microscope, and quantification was performed using ImageJ software (version 1.52; National Institutes of Health, Bethesda, MD, USA) [14].

### 2.8. Immunohistochemistry (IHC)

Paraffin-embedded tissue sections were deparaffinized in xylene and rehydrated through a graded ethanol series. After rinsing with PBS, the sections were blocked with 5% bovine serum albumin (BSA) at room temperature for 30 min. Sections were incubated with a polyclonal antibody against *MFAP2* (1:500; cat. no. UPAC618Mu01; Cloud-Clone Corp., Wuhan, China) overnight at 4 °C. The following day, sections were processed using a universal two-step detection kit (Mouse/Rabbit Enhanced Polymer Detection System; Zhongshan Golden Bridge Biotechnology Co., Ltd., Beijing, China). Images were acquired and analyzed using the Leica Application Suite X (LAS X; Leica Microsystems GmbH, Wetzlar, Germany) [15].

### 2.9. Western Blot Analysis

Proteins were extracted from cells or tissues using RIPA lysis buffer (Beyotime Institute of Biotechnology, Shanghai, China), followed by homogenization and centrifugation at 12,000× *g* for 15 min at 4 °C. Protein concentrations were determined using a BCA assay kit (Glpbio, Montclair, CA, USA, catalogue number GK10009). Equal amounts of protein (30 µg) were separated on 10% SDS-PAGE gels and transferred onto PVDF membranes (MilliporeSigma, Burlington, MA, USA). When analyzing multiple proteins from the same lysate, the membranes were cut horizontally based on the predicted molecular weights of the target proteins prior to blocking. In cases where target proteins possessed similar molecular weights, membranes were alternatively subjected to sequential stripping and re-probing. The cut or intact membranes were blocked with 5% non-fat milk for 1 h and then incubated with primary antibodies overnight at 4 °C. The primary antibodies used included anti-*MFAP2* (1:1000; Cloud-Clone Corp.), anti-ATG7 (1:1000; cat. no. 10088-2-AP), anti-ATG5 (1:1000; cat. no. 10181-2-AP), anti-Beclin 1 (1:1000; cat. no. 11306-1-AP), anti-p62/SQSTM1 (1:1000; cat. no. 18420-1-AP), anti-LC3B (1:1000; cat. no. 18725-1-AP), anti-β-catenin (1:1000; cat. no. 51067-2-AP), anti-MMP9 (1:1000; cat. no. 10375-2-AP), anti-c-Myc (1:1000; cat. no. 10828-1-AP), and anti-Cyclin D1 (1:1000; cat. no. 26903-1-AP) (all from Proteintech Group, Inc., Rosemont, IL, USA). After three washes with TBST, the membranes were incubated with HRP-conjugated anti-mouse or anti-rabbit IgG secondary antibodies (1:5000; cat. nos. SA00001-1 and SA00001-2; Proteintech Group, Inc.) at room temperature for 1 h. Protein bands were visualized using an ECL chemiluminescence detection system (Thermo Fisher Scientific, Inc.) [8].

### 2.10. Animal Experiments

Female wild-type C57BL/6J mice (7–8 weeks old; weight, 20 ± 2 g) were purchased from Liaoning Changsheng Biotechnology Co., Ltd. (Liaoning, China). These female cohorts were specifically chosen to minimize behavioral confounders—such as aggression and territorial fighting frequently observed in group-housed male mice—thereby reducing unnecessary physiological stress and ensuring more consistent in vivo outcomes. Mice were housed in a specific pathogen-free environment with a 12-h light/dark cycle and ad libitum access to food and water. An orthotopic glioblastoma model was established using the murine GL261 glioblastoma cell line. The mice were randomly assigned to three groups (*n* = 6 per group): the sham-operated control group (Control), the tumor model group (Model), and the *MFAP2*-knockdown group (sh-*MFAP2*), in which cells were stably transduced with sh-*MFAP2* lentiviral particles (Shanghai GeneChem Co., Ltd., Shanghai, China). Although a formal a priori statistical power analysis was not conducted, this sample size was determined empirically based on well-established protocols from comparable orthotopic GL261 studies. This approach ensured sufficient biological replication to detect meaningful therapeutic differences while strictly adhering to the 3Rs principles of animal research to minimize animal use. Anesthesia was induced via intraperitoneal injection of sodium pentobarbital (0.75%, 50 mg/kg). Mice were secured in a stereotaxic frame. A burr hole was drilled 0.15 mm posterior and 2.0 mm lateral to the bregma. A 10-µL microsyringe was used to inject 10 µL of GL261 cell suspension (5 × 10^5^ cells) at a depth of 4 mm. The injection was performed over 2 min, followed by a 3-min stabilization period. The incision was sutured, and mice were monitored daily for body weight and neurological signs. All animal experiments were approved by the Ethics Committee of the Second Affiliated Hospital of Zhengzhou University (Approval No. KY2025120) and performed in accordance with the National Institutes of Health Guide for the Care and Use of Laboratory Animals.

### 2.11. Statistical Analysis

All statistical analyses were performed using SPSS software (version 25.0; IBM Corp., Armonk, NY, USA). The Shapiro–Wilk test was used to assess the normality of the data when the sample size was sufficient. For normally distributed data, comparisons between two groups were conducted using the Student’s *t*-test, while multiple-group comparisons were analyzed using one-way analysis of variance (ANOVA) followed by Tukey’s post hoc test. For data involving small sample sizes (such as small paired clinical cohorts) where normal distribution could not be robustly confirmed, appropriate non-parametric tests, such as the Wilcoxon signed-rank test, were employed. Data are presented as the mean ± standard deviation (SD). A *p*-value < 0.05 was considered to indicate a statistically significant difference [8].

### 2.12. Experimental Design, Replicates, Randomization, Blinding, and Sample Size

For in vitro experiments, all assays were performed in at least three independent biological replicates (independent cell preparations on different days). For CCK-8 assays, each condition was additionally measured in technical replicate wells per experiment. For wound-healing and Transwell assays, random microscopic fields per insert were quantified, and the mean value per insert was used as one biological replicate. For Western blot densitometry using cell lysates, protein levels were quantified from at least three independent experiments. For animal studies, mice were randomly assigned to groups prior to tumor implantation, and analyses were performed according to prespecified endpoints. Sample size (*n* = 6 per group) was determined based on prior orthotopic GL261 studies employing similar endpoints and effect sizes, a formal a priori power analysis was not performed; this has been acknowledged as a limitation. Animal experiments were reported in accordance with ARRIVE 2.0 recommendations.

## 3. Results

### 3.1. MFAP2 Is Upregulated in Glioblastoma and Is Associated with Poor Prognosis

**Figure 1:**
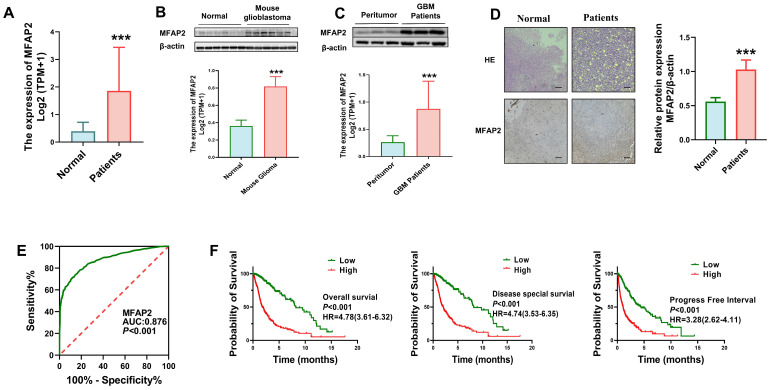
***MFAP2* expression is upregulated in GBM and correlates with poor prognosis in patients with GBM.** (**A**) Differential *MFAP2* expression in normal tissues (GTEx, *n* = 1152) and GBM tissues (TCGA, *n* = 689). *** *p* < 0.001. (**B**) Western blot analysis showing *MFAP2* protein expression differences between mouse glioblastoma tissues and adjacent non-tumor tissues (*n* = 6). *** *p* < 0.001. (**C**) Representative Western blot images and corresponding quantitative analysis of *MFAP2* protein levels in human glioblastoma tissues versus paired adjacent non-tumor tissues. While representative blots from 3 patients are shown, the quantitative statistical analysis was performed on a total cohort of 6 paired samples (*n* = 6). Statistical significance was evaluated using the non-parametric Wilcoxon signed-rank test. *** *p* < 0.001. (**D**) Immunohistochemical images showing elevated *MFAP2* expression in glioblastoma patient tissues (*n* = 3). *** *p* < 0.001, (Scale bar, 50 μm). (**E**) ROC curve of *MFAP2* expression distinguishing TCGA-GBM tumors (*n* = 706) from GTEx normal brain tissues (*n* = 1152), (AUC = 0.876, *p* < 0.001). (**F**) *MFAP2* expression is elevated in deceased glioblastoma patients compared with survivors.

To investigate the role of *MFAP2* in GBM, we first assessed *MFAP2* expression patterns and found it to be significantly elevated in GBM relative to normal brain tissues (Figure 1A). Subsequent validation in an orthotopic mouse glioblastoma model revealed *MFAP2* protein levels 2.3–fold higher in tumor tissues than in normal brain tissues (Figure 1B). Parallel analyses of human clinical specimens showed a 3.3–fold increase in *MFAP2* protein expression in GBM tissues compared to matched adjacent peritumoral (Figure 1C).

Immunohistochemical staining confirmed robust *MFAP2* localization in the membrane and cytoplasm of GBM cells, with minimal to no expression detected in peritumoral (Figure 1D). Receiver operating characteristic (ROC) curve analysis yielded an area under the curve (AUC) of 0.876, indicating strong diagnostic potential for distinguishing GBM from normal brain tissues (Figure 1E). Survival analyses further demonstrated that high *MFAP2* expression correlated significantly with shorter overall survival (OS), disease-specific survival (DSS), and progression-free survival (PFS) in GBM patients (Figure 1F), suggesting *MFAP2* as a promising prognostic biomarker for poor clinical outcomes. Furthermore, clinicopathological analysis revealed that high MFAP2 expression was significantly associated with advanced WHO grade and IDH wild-type status (Supplementary Table S1).

### 3.2. Effects of MFAP2 Expression on the Viability, Apoptosis, Migration and Invasion of Glioblastoma Cells

**Figure 2:**
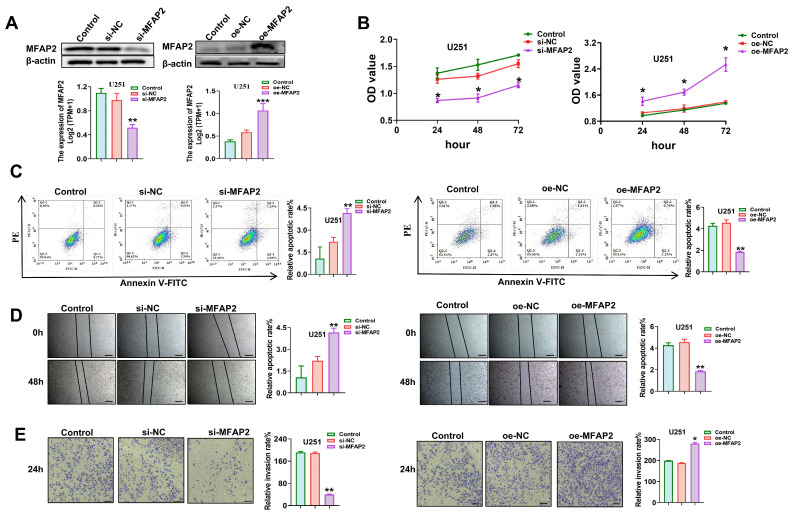
**Effects of *MFAP2* expression on U251 cells viability, apoptosis, migration and invasion.** (**A**) Validation of *MFAP2* knockdown and overexpression efficiency in U251 cells by Western blotting. Band intensities were quantified using ImageJ (** *p* < 0.01, *** *p* < 0.001), with the control group serving as the baseline. (**B**) Viability of U251 cells with *MFAP2* knockdown or overexpression (* *p* < 0.05 vs. Control). (**C**) Apoptosis of U251 cells under *MFAP2* knockdown or overexpression conditions (** *p* < 0.01 vs. Control). (**D**) Migration of U251 cells following *MFAP2* knockdown or overexpression. Scale bar = 200 μm. (** *p* < 0.01 vs. Control). (**E**) Invasion of U251 cells after *MFAP2* knockdown or overexpression. Magnification, ×200; Scale bar = 100 μm. (* *p* < 0.05, ** *p* < 0.01 vs. Control).

Prior to investigating the functional consequences of *MFAP2* manipulation, we first evaluated its baseline protein expression across a panel of representative cross-species glioblastoma cell lines. Western blot analysis confirmed that *MFAP2* is constitutively and robustly expressed at comparable high levels in human (U251 and U87MG), murine (GL261), and rat (C6) glioblastoma cells (Supplementary Figure S1A). This conserved expression profile provided a strong rationale for utilizing these specific cell lines for our in vitro assays and established a reliable basis for employing the syngeneic GL261 model in subsequent in vivo studies. To dissect the functional role of *MFAP2* in GBM pathogenesis, we engineered U251 cells with stable *MFAP2* knockdown or overexpression, with manipulation efficiency confirmed by Western blotting (Figure 2A). Functional assays revealed that *MFAP2* silencing suppressed U251 cell viability by 42.7% relative to controls, whereas *MFAP2* overexpression enhanced cell proliferation by 58.3% (Figure 2B). Correspondingly, flow cytometry analysis demonstrated a 3.1-fold increase in apoptotic rate in *MFAP2*-silenced cells, while overexpression reduced apoptosis by 61.2% compared to empty vector controls (Figure 2C).

Wound healing assays showed that *MFAP2* knockdown decreased cell migration rate by 51.4% at 24 h post-scratching, whereas overexpression accelerated wound closure by 47.2% within the same timeframe (Figure 2D). Transwell invasion assays further validated these pro-tumorigenic effects, with *MFAP2* knockdown reducing invasive cell counts by 58.7% and overexpression increasing invasion by 63.9% relative to controls (Figure 2E). To ensure that these phenotypic alterations were not restricted to a single cell line, we performed parallel functional validations using a second human GBM cell line, U87MG. Consistent with the trends observed in U251 cells, the manipulation of *MFAP2* expression in U87MG cells elicited similar and significant regulatory effects on cell viability, apoptosis, migration, and invasion (Supplementary Figure S1B–F). Taken together, these mechanistic studies across multiple GBM cell models establish *MFAP2* as a potent oncogenic driver that enhances GBM cell proliferation, survival, migration, and invasion.

### 3.3. MFAP2 Modulates the Biological Behavior of U251 Cells by Regulating Autophagy

**Figure 3:**
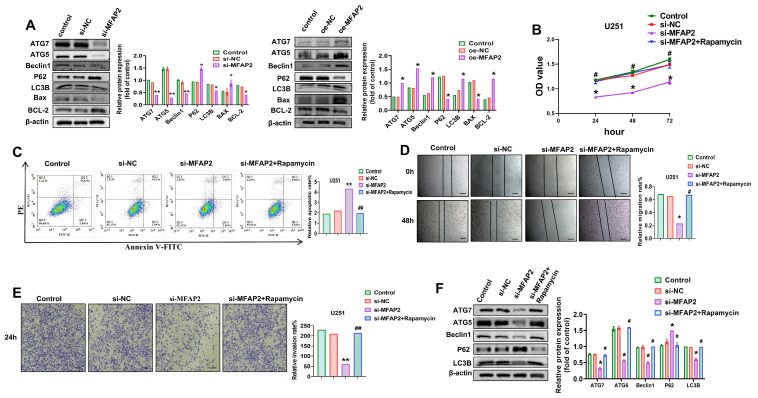
***MFAP2* affects U251 cells viability, apoptosis, migration and invasion by inhibiting autophagy.** (**A**) Levels of autophagy- and apoptosis-related proteins in U251 cells after *MFAP2* knockdown or overexpression. Band intensities were quantified using ImageJ (* *p* < 0.05, ** *p* < 0.01 vs. Control). (**B**) Viability of U251 cells after *MFAP2* knockdown with rapamycin treatment (* *p* < 0.05 vs. Control, ^#^ *p* < 0.05 vs. si-*MFAP2*). (**C**) Apoptosis of U251 cells following *MFAP2* knockdown combined with rapamycin treatment (** *p* < 0.01 vs. Control, ^##^ *p* < 0.01 vs. si-*MFAP2*). (**D**) Migration of U251 cells after *MFAP2* knockdown and rapamycin treatment. Scale bar = 200 μm. (* *p* < 0.05 vs. Control, ^#^ *p* < 0.05 vs. si-*MFAP2*). (**E**) Invasion of U251 cells following *MFAP2* knockdown and rapamycin treatment. Magnification, ×200; Scale bar = 100 μm. (** *p* < 0.01 vs. Control, ^##^ *p* < 0.01 vs. si-*MFAP2*). (**F**) Levels of autophagy-related proteins in U251 cells after *MFAP2* knockdown with rapamycin treatment. Band intensities were quantified using ImageJ (* *p* < 0.05 vs. Control, ^#^ *p* < 0.05 vs. si-*MFAP2*).

Given the critical role of autophagy in tumor progression, we next explored whether *MFAP2* modulates autophagic activity in GBM cells. *MFAP2* knockdown in U251 cells reduced protein levels of key autophagy regulators (ATG7, ATG5, LC3B, and Beclin 1) by 41.2–58.7% and increased p62 accumulation by 2.7–fold, consistent with impaired autophagic flux; conversely, *MFAP2* overexpression upregulated these autophagy markers by 52.3–71.5% and decreased p62 levels by 63.1% (Figure 3A). *MFAP2* silencing also shifted the Bax/Bcl-2 ratio pro-apoptotically, with a 3.4-fold increase relative to controls.

To establish a functional link between *MFAP2*-mediated autophagy and the malignant phenotypes of U251 cells, we treated *MFAP2*-silenced cells with the autophagy activator rapamycin (100 nM). Rapamycin treatment restored cell viability by 57.2% (Figure 3B) and reduced apoptosis by 62.4% (Figure 3C) in *MFAP2*-knockdown cells, while also rescuing migratory capacity by 48.9% and invasive potential by 53.7% (Figure 3D,E). Western blotting confirmed that rapamycin reversed the suppression of autophagy markers in *MFAP2*-silenced cells, restoring ATG7, ATG5, LC3B, and Beclin 1 levels and reducing p62 accumulation (Figure 3F). Collectively, these findings demonstrate that *MFAP2* promotes the aggressive behavior of U251 cells at least in part by enhancing autophagic flux.

### 3.4. MFAP2 Regulates the Biological Behavior of U251 Cells by Activating the Wnt/β-Catenin Signaling Pathway via Autophagy

**Figure 4:**
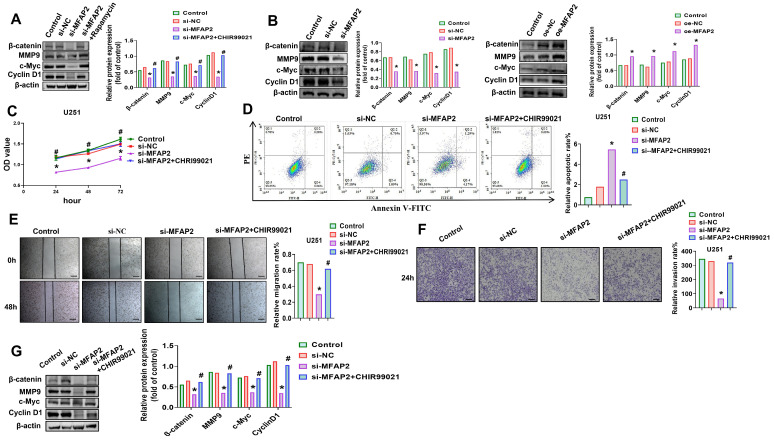
***MFAP2* regulates U251 cells viability, proliferation, apoptosis, migration and invasion through modulation of the Wnt/β-catenin signaling pathway.** (**A**) Levels of β-catenin and its downstream effectors in U251 cells after *MFAP2* knockdown and rapamycin treatment. Band intensities were quantified using ImageJ (* *p* < 0.05 vs. Control, ^#^ *p* < 0.05 vs. si-*MFAP2*). (**B**) Levels of β-catenin and downstream proteins in U251 cells following *MFAP2* knockdown or overexpression. Band intensities were quantified using ImageJ (* *p* < 0.05 vs. Control). (**C**) Viability of U251 cells after *MFAP2* knockdown with or without CHIR99021 treatment (* *p* < 0.05 vs. Control, ^#^ *p* < 0.05 vs. si-*MFAP2*). (**D**) Apoptosis of U251 cells following *MFAP2* knockdown and CHIR99021 treatment (* *p* < 0.05 vs. Control, ^#^ *p* < 0.05 vs. si-*MFAP2*). (**E**) Migration of U251 cells after *MFAP2* knockdown and CHIR99021 treatment. Scale bar = 200 μm. (* *p* < 0.05 vs. Control, ^#^ *p* < 0.05 vs. si-*MFAP2*). (**F**) Invasion of U251 cells following *MFAP2* knockdown combined with CHIR99021 treatment. Magnification, ×200; Scale bar = 100 μm. (* *p* < 0.05 vs. Control, ^#^ *p* < 0.05 vs. si-*MFAP2*). (**G**) Levels of β-catenin and downstream proteins in U251 cells after *MFAP2* knockdown and CHIR99021 treatment. Band intensities were quantified using ImageJ (* *p* < 0.05 vs. Control, ^#^ *p* < 0.05 vs. si-*MFAP2*).

To delineate the downstream signaling cascade mediating *MFAP2*’s oncogenic effects, we focused on the Wnt/β-catenin pathway, a key regulator of GBM progression. *MFAP2* knockdown in U251 cells reduced β-catenin protein levels by 56.3% and suppressed its downstream targets MMP9, c-Myc, and Cyclin D1 by 48.7–62.1% (Figure 4A). Notably, autophagy reactivation with rapamycin restored β-catenin and its effector proteins to 89.2–94.7% of control levels in *MFAP2*-silenced cells (Figure 4A). Reciprocal manipulation of *MFAP2* expression confirmed this regulatory relationship: overexpression increased β-catenin, MMP9, c-Myc, and Cyclin D1 by 53.8–72.4%, while knockdown suppressed these proteins by 45.2–61.3% (Figure 4B).

To validate the Wnt/β-catenin pathway as a functional effector of *MFAP2*, we treated *MFAP2*-silenced cells with the GSK3β inhibitor CHIR99021 (6 µM), a potent Wnt agonist. CHIR99021 treatment rescued cell viability by 59.4% (Figure 4C), reduced apoptosis by 63.7% (Figure 4D), restored migratory capacity by 51.2% (Figure 4E), and recovered invasive potential by 54.8% (Figure 4F) in *MFAP2*-knockdown cells. Western blotting confirmed that CHIR99021 reversed the suppression of β-catenin and its downstream targets in *MFAP2*-silenced cells (Figure 4G). Collectively, these mechanistic studies establish that *MFAP2* drives the malignancy of U251 cells through an autophagy-dependent activation of the Wnt/β-catenin signaling cascade.

### 3.5. Downregulation of MFAP2 Inhibits GBM Tumor Growth In Vivo

**Figure 5:**
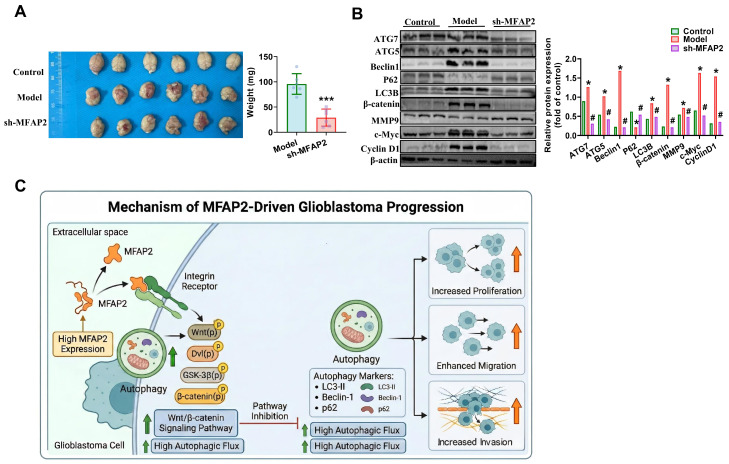
**Knockdown of *MFAP2* suppresses GBM tumor growth in vivo.** (**A**) Tumor volumes in female C57BL/6J mice 21 days after orthotopic implantation of GL261 cells (*n* = 6). Statistical analysis of body weight after orthotopic implantation (*** *p* < 0.001 vs. Control). (**B**) Protein expression levels of autophagy-related factors and components of the Wnt/β-catenin signaling pathway. Band intensities were quantified using ImageJ (* *p* < 0.05 vs. Control, ^#^ *p* < 0.05 vs. Model). (**C**) A schematic diagram showing that *MFAP2* can influence GBM progression via its autophagic regulation of Wnt/β-catenin signaling.

To validate our in vitro findings in a physiological context, we established an orthotopic mouse glioblastoma model by intracranial injection of GL261 cells stably transduced with sh-*MFAP2* or control vectors. Mice bearing *MFAP2*-silenced tumors exhibited 62.4% smaller mean tumor volumes and 58.7% lower mean tumor weights (Figure 5A) compared with control groups at endpoint analysis. Immunoblotting of tumor tissues confirmed that *MFAP2* knockdown in vivo reduced protein levels of autophagy regulators (ATG7, ATG5, LC3B, and Beclin 1) by 43.2–59.1% and Wnt pathway components (β-catenin, c-Myc, and Cyclin D1) by 47.6–61.3% (Figure 5B), recapitulating our in vitro observations. Collectively, our findings establish that the Wnt/β-catenin signaling pathway mediates the effects of *MFAP2* on GBM cell proliferation and migration, a process further modulated by *MFAP2*-induced autophagy. These results identify *MFAP2* as a positive regulator of both autophagy and Wnt/β-catenin signaling in GBM. Crucially, *MFAP2* promotes GBM progression by orchestrating a functional crosstalk where it modulates the Wnt/β-catenin axis via the autophagic pathway (Figure 5C).

## 4. Discussion

Glioblastoma (GBM) remains the most aggressive and lethal primary brain tumor in adults, defined by relentless proliferation, diffuse invasiveness, and profound resistance to conventional therapy [1]. Despite incremental advances in surgical resection, radiotherapy, and targeted pharmacotherapy, median overall survival for GBM patients hovers at just 15–16 months, highlighting the urgent need to decode the molecular drivers of this devastating disease [2]. In this study, we identify microfibrillar-associated protein 2 (*MFAP2*) as a novel oncogenic driver in GBM and demonstrate that elevated *MFAP2* expression promotes GBM malignancy by activating an autophagy-dependent Wnt/β-catenin signaling axis. These findings not only expand our understanding of GBM pathogenesis but also establish *MFAP2* as a promising prognostic biomarker and therapeutic target.

Our study reveals that *MFAP2* is significantly upregulated in human GBM tissues compared with adjacent peritumoral brain tissue, a finding that strongly correlates with poor clinical outcomes. While previous studies have documented *MFAP2* overexpression in gastric cancer, hepatocellular carcinoma, and melanoma [5,6,7], our functional experiments establish that *MFAP2* is not merely a structural byproduct of tumor remodeling, but an active orchestrator of GBM aggressiveness. By robustly promoting cell viability and invasive capacity while conferring apoptotic resistance, *MFAP2* drives a profound pro-tumorigenic phenotypic shift. These observations align with the emerging paradigm that specific extracellular matrix (ECM) glycoproteins actively fuel epithelial–mesenchymal transition (EMT)-like programs and tumor invasiveness [7,8], firmly positioning *MFAP2* as an essential structural and functional component of the GBM microenvironment.

A key conceptual advance of this study is the identification of *MFAP2* as an upstream positive regulator of autophagic flux. Basal autophagy is a homeostatic process essential for the renewal of intracellular organelles. However, autophagy in cancer is notoriously context-dependent. In highly malignant tumors like GBM, accelerated autophagic flux serves as a cytoprotective adaptive mechanism. By recycling intracellular components, hyperactive autophagy fulfills the heightened metabolic demands of rapidly proliferating cells, mitigates the stress associated with matrix detachment, and thereby actively fuels tumor cell migration and invasion [11,16]. By demonstrating that *MFAP2* depletion impairs autophagic flux—and that pharmacological reactivation of autophagy rescues the aggressive phenotypes of *MFAP2*-silenced cells—we provide compelling evidence that *MFAP2* exploits this highly conserved metabolic pathway to sustain tumor survival. Importantly, these results expand the traditional understanding of tumor biology by illustrating an “outside–in” signaling paradigm, where an ECM-associated molecule directly modulates intracellular autophagic machinery [17].

Building upon this mechanism, we further elucidated a downstream link between *MFAP2*-driven autophagy and the canonical Wnt/β-catenin cascade, a pathway notoriously implicated in GBM stemness and progression [9,10]. Our data indicate that the stabilization and activation of β-catenin, along with its downstream effectors (MMP9, c-Myc, Cyclin D1), are fundamentally dependent on *MFAP2*-mediated autophagic activity. Mechanistically, this is consistent with recent findings suggesting that autophagy can prevent β-catenin degradation by regulating GSK3β dynamics [18,19]. The fact that Wnt reactivation (via GSK3β inhibition) bypassed the suppressive effects of *MFAP2* knockdown robustly validates this hierarchical signaling axis. A similar regulatory network was recently observed in oral squamous cell carcinoma [8], implying that the *MFAP2*-autophagy-Wnt axis may represent a evolutionarily conserved oncogenic program across various solid tumors.

Beyond the classical malignant phenotypes of proliferation and invasion, emerging evidence indicates that oncogenic signaling networks, including the canonical Wnt/β-catenin cascade, may profoundly modulate membrane excitability and ion channel expression in GBM. For instance, recent studies have highlighted the critical involvement of voltage-gated sodium channels (VGSCs) in maintaining GBM stemness, promoting cell cycle progression, and driving therapeutic resistance [20]. Furthermore, compartment-specific ionic dynamics, such as those regulated by the sodium-calcium exchanger (NCX), have been shown to be functionally indispensable for lamellipodia formation and the highly invasive strategies of GBM cells [21]. Although our current study primarily focused on conventional phenotypic assays, it is highly plausible that the *MFAP2*-driven activation of Wnt/β-catenin signaling may orchestrate these broader electrophysiological and ion transport programs. Investigating whether *MFAP2* signaling intersects with VGSC- and NCX-mediated pathways to modulate tumor stemness and membrane excitability represents a compelling frontier for future research, which could uncover novel synergistic therapeutic vulnerabilities in glioblastoma.

The clinical and biological implications of these findings are substantial. ROC analysis revealed that *MFAP2* possesses strong discriminatory power (AUC = 0.876) for distinguishing GBM from adjacent peritumoral tissues, consistent with its reported prognostic value in other tumors [6]. As an ECM-associated microfibrillar protein (also known as MAGP-1), the precise cellular origin of *MFAP2* within the complex GBM microenvironment warrants further elucidation. While our immunohistochemical observations indicate prominent expression within tumor cells, the contribution of stromal compartments—such as activated astrocytes, endothelial cells, or immune infiltrates—to *MFAP2* deposition cannot be definitively excluded without future single-cell transcriptomic or spatial profiling studies. Within the ECM, *MFAP2* traditionally interacts with elastic fibers and regulates the bioavailability of signaling molecules, such as TGF-β [22,23]. In the context of GBM, this abnormal ECM deposition may alter matrix stiffness, thereby amplifying mechanotransduction processes and integrin signaling known to drive tumor aggressiveness [24,25]. Targeting *MFAP2* could therefore simultaneously impair autophagy, inhibit Wnt/β-catenin signaling, and weaken pro-invasive microenvironmental cues, representing a multi-layered therapeutic strategy. This is particularly promising given the limited success of single-agent therapies in GBM.

Despite these strengths, our study has several limitations that warrant future investigation. First, our in vivo studies exclusively utilized female mice to minimize behavioral confounders (such as aggression-induced stress) associated with group-housed males. However, recognizing sex as a crucial biological variable in glioblastoma pathogenesis and treatment response, the lack of male cohorts limits our ability to evaluate potential sex-based differences. Future investigations must incorporate both sexes to confirm the broad applicability of these findings. Second, regarding experimental design, our animal sample size (*n* = 6) was determined empirically based on established literature rather than formal a priori statistical power calculations, representing a methodological limitation. Third, our clinical validation utilized adjacent peritumoral tissues as baseline controls. We fully acknowledge that the peritumoral region often harbors underlying molecular, genetic, or microenvironmental alterations due to field cancerization and tumor infiltration, and therefore cannot perfectly represent truly healthy “normal” brain tissue. Future validations utilizing larger clinical cohorts, ideally incorporating true healthy brain tissue controls (e.g., from trauma patients), are necessary to rigorously confirm our observations. Fourth, while we demonstrate that *MFAP2* modulates autophagy and Wnt/β-catenin signaling, the specific molecular intermediates (e.g., integrins or upstream regulators of mTOR and ULK1) linking *MFAP2* to these pathways remain undefined. Fifth, given the marked molecular heterogeneity of GBM, evaluating *MFAP2* in patient-derived xenograft (PDX) and organoid models across different GBM subtypes will be essential to fully establish its translational potential and subtype-specific significance. Sixth, our evaluation of cell death was primarily based on in vitro flow cytometric assays, which capture apoptotic events. However, in the complex in vivo microenvironment of GBM, profound hypoxia and vascular abnormalities predominantly drive necrosis rather than classic apoptosis [26]. Thus, the precise relationship between *MFAP2*, tumor growth acceleration, and specific modes of cell death in vivo requires further pathological investigation. Furthermore, our current assessment of autophagy relied on the steady-state expression of autophagy-related markers. Future studies incorporating dynamic flux-based assays, such as lysosomal inhibition or tandem fluorescent reporters, are necessary to definitively quantify autophagic activity. Finally, our current experimental design did not evaluate the impact of standard-of-care therapies, such as temozolomide (TMZ) chemotherapy or radiotherapy, on *MFAP2* production. Treatment-induced cellular senescence and ECM remodeling are known to profoundly alter the glioblastoma microenvironment [27,28]. Investigating whether chemo-radiotherapy dynamically modulates *MFAP2* expression, and how this modulation influences treatment resistance, remains a critical direction for our future studies.

In conclusion, this study identifies *MFAP2* as a novel oncogenic regulator in GBM and reveals a previously unrecognized *MFAP2*-autophagy-Wnt/β-catenin axis that drives tumor progression. By linking ECM components to intracellular autophagy and canonical Wnt signaling, our findings provide new insights into GBM biology. Given the preclinical nature of our study and the limited clinical sample size, we conservatively propose that *MFAP2* serves as a candidate prognostic biomarker and a potential biological vulnerability in GBM, rather than an established therapeutic target. Extensive translational validations in broader clinical cohorts and advanced patient-derived models are required before the therapeutic relevance of targeting *MFAP2* can be firmly established.

## Data Availability

The datasets used and/or analyzed during the current study are available from the corresponding author on reasonable request. The datasets generated and/or analyzed during the current study are available in the TCGA repository, [https://portal.gdc.cancer.gov], accessed on 17 April 2025, and GTEx repository [https://gtexportal.org], accessed on 17 April 2025.

## Supplementary Materials

The following supporting information can be downloaded at: https://www.mdpi.com/article/10.3390/biomedicines14051003/s1, Figure S1: Effects of MFAP2 expression on U87 cells viability, apoptosis, migration and invasion; Table S1: Clinical and molecular characteristics of the patient cohort based on MFAP2 expression.
