# Supplementary material for: MFAP2 Promotes Glioblastoma Malignant Phenotypes via Autophagy-Dependent Activation of Wnt/β-Catenin Signaling
Source: Biomedicines. 2026 Apr 28;14(5):1003. doi: 10.3390/biomedicines14051003 (PMC13203895; doi:10.3390/biomedicines14051003)
Supplement: Supplementary file 1 [file biomedicines-14-01003-s001.zip › Supplementary Figure S1.pdf]

SupplementaryFigure S1

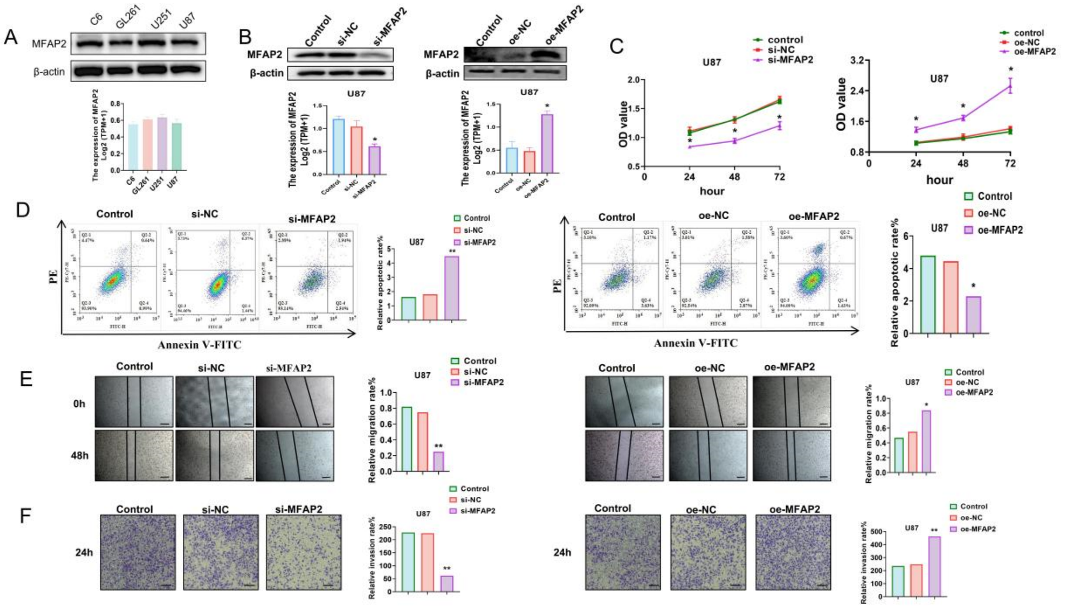

Figure S1. Effects of MFAP2 expression on U87 cells viability, apoptosis, migration and invasion. (A) The level of MFAP2 was evaluated in various GBM cell lines by WB. (B) Validation of MFAP2 knockdown and overexpression efficiency in U87 cells by Western blotting. Band intensities were quantified using ImageJ ($^{*}P$<0.05), with the control group serving as the baseline. (C) Viability of U87 cells with MFAP2 knockdown or overexpression ($^{*}P$<0.05 vs. Control). (D) Apoptosis of U87 cells under MFAP2 knockdown or overexpression conditions ($^{*}P$<0.05, $^{**}P$<0.01 vs. Control). (E) Migration of U87 cells following MFAP2 knockdown or overexpression. Scale bar = 200 μm. ($^{*}P$<0.05, $^{**}P$<0.01 vs. Control). (F) Invasion of U87 cells after MFAP2 knockdown or overexpression. Magnification, ×200; Scale bar = 100 μm. ($^{**}P$<0.01 vs. Control).
