# Supplementary material for: MFAP2 Promotes Glioblastoma Malignant Phenotypes via Autophagy-Dependent Activation of Wnt/β-Catenin Signaling
Source: Biomedicines. 2026 Apr 28;14(5):1003. doi: 10.3390/biomedicines14051003 (PMC13203895; doi:10.3390/biomedicines14051003)
Supplement: Supplementary file 1 [file biomedicines-14-01003-s001.zip › Supplementary Table S1.pdf]

Characteristics	Low expression of MFAP2	High expression of MFAP2	P value
n	349	350	
WHO grade, n (%)			< 0.001
G2	174 (27.3%)	50 (7.8%)	
G3	129 (20.3%)	116 (18.2%)	
G4	4 (0.6%)	164 (25.7%)	
IDH status, n (%)			< 0.001
WT	34 (4.9%)	212 (30.8%)	
Mut	312 (45.3%)	131 (19%)	

Supplementary Table S1. Clinical and molecular characteristics of the patient cohort based on MFAP2 expression. The table summarizes the distribution of World Health Organization (WHO) grades (G2, G3, G4) and Isocitrate Dehydrogenase (IDH) mutation status across patients with low (n = 349) and high (n = 350) MFAP2 expression. Statistical significance between the two expression groups was determined using the Pearson's Chi-square test (or Fisher's exact test, where appropriate). $P < 0.05$ was considered statistically significant. Abbreviations: MFAP2, microfibrillar-associated protein 2; WHO, World Health Organization; IDH, isocitrate dehydrogenase; WT, wildtype; Mut, mutant; n, number of patients.
