# Supplementary figures and images for: MFAP2 Promotes Glioblastoma Malignant Phenotypes via Autophagy-Dependent Activation of Wnt/β-Catenin Signaling
Source: Biomedicines. 2026 Apr 28;14(5):1003. doi: 10.3390/biomedicines14051003 (PMC13203895; doi:10.3390/biomedicines14051003)

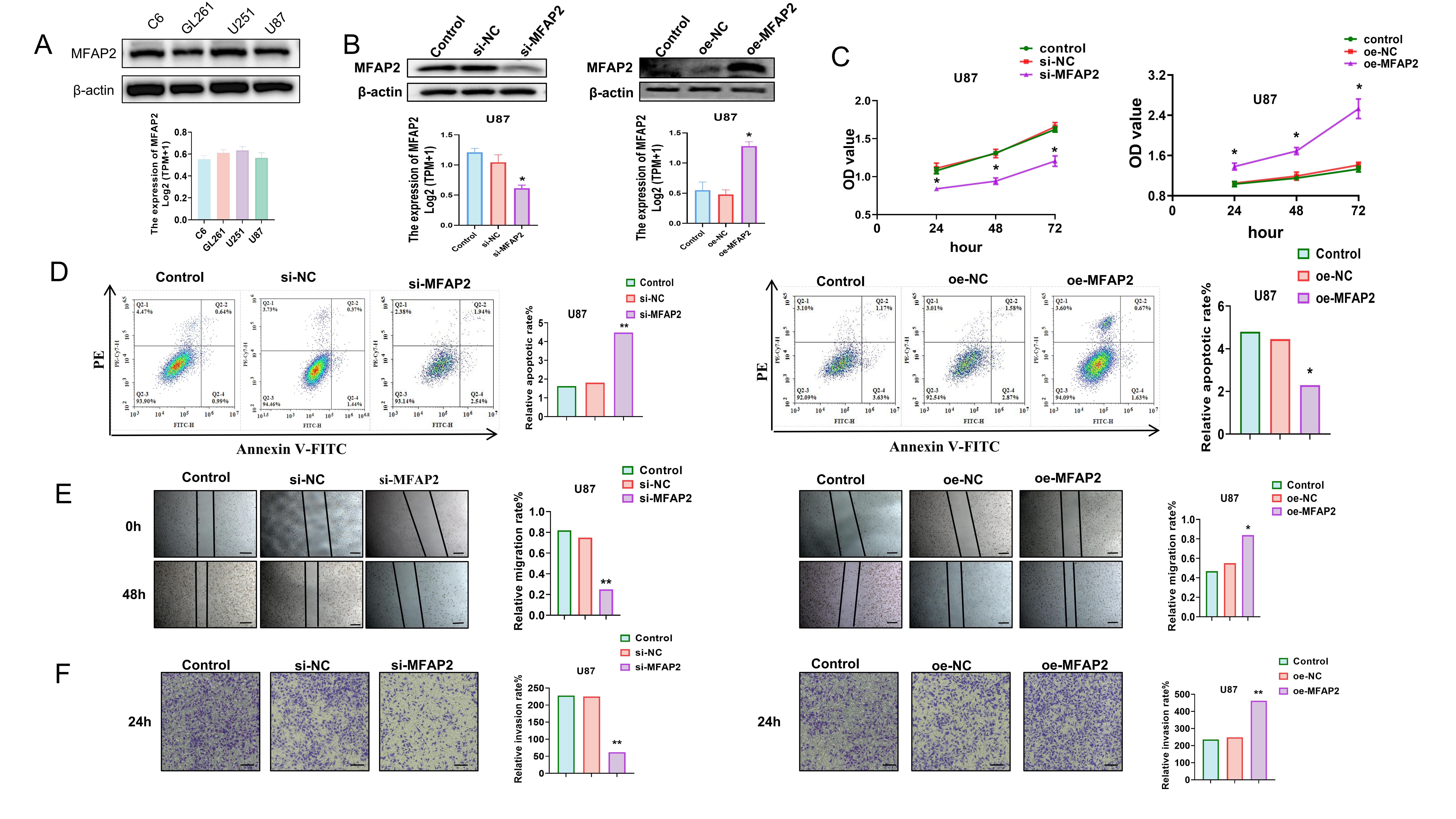

Supplement: Supplementary file 1 [file biomedicines-14-01003-s001.zip › Supplementary Figure S1.jpg]
